# Association of frailty and functional recovery in an Acute Care for Elders unit: a prospective observational study

**DOI:** 10.1186/s12877-022-03290-2

**Published:** 2022-07-21

**Authors:** Hsiao-Chen Chang, Yi-Yen Lu, Sheng-Lun Kao

**Affiliations:** 1 Department of Family Medicine, Hualien Tzu Chi Hospital, Buddhist Tzu Chi Medical Foundation, No. 707, Sec. 3, Chung Yang Rd, Hualien, 97002 Taiwan; 2 Department of Nursing, Hualien Tzu Chi Hospital, Buddhist Tzu Chi Medical Foundation, Hualien, Taiwan; 3 Department of Family Medicine, Tzu Chi University, Hualien, Taiwan; 4 Institute of Medical Sciences, Tzu Chi University, Hualien, Taiwan

**Keywords:** Frailty, FRAIL scale, Functional decline, Activities of daily living, Acute Care for Elders unit

## Abstract

**Background:**

Evidence on the effects of Acute Care for Elders (ACE) units in frail older adults remains limited. Therefore, we aimed to evaluate the effects of the ACE unit on functional outcomes in frail older adults.

**Methods:**

In this prospective observational study, we enrolled 114 consecutive patients aged 65 years and older admitted to the ACE unit for acute medical conditions between October 2019 and September 2020. The FRAIL scale (5-question assessment of fatigue, resistance, aerobic capacity, illnesses, and loss of weight) was used to classify the patients into three groups: robust (score = 0, *n* = 28), prefrail (score = 1–2, *n* = 57), and frail (score = 3–5, *n* = 29). The primary outcome was the activities of daily living (ADL) measured by the Barthel Index at admission and before discharge. Paired sample t-test was employed to determine the difference in ADL. Multiple linear regression analysis, with adjustment for covariates, was conducted to examine the association between frailty status and change in ADL.

**Results:**

Among 114 patients enrolled (mean age, 79.8 ± 8.1 years; mean length of stay, 6.4 ± 5.6 days), 77 (67.5%) were female. ADL at admission (60.3 ± 31.9) and before discharge (83.7 ± 21.6) were significantly different (*P* < 0.001). After covariates adjustment, a significant association between frailty status and change in ADL was found (prefrail vs. robust: β = 9.0, 95% confidence interval [CI] 0.3–17.6, *P* = 0.04; frail vs. robust: β = 13.4, 95% CI 2.7–24.0, *P* = 0.01).

**Conclusions:**

Older adults with frailty experienced functional improvement after admission to the ACE unit. Prefrail and frail groups were associated with a more significant change in ADL between admission and discharge compared to the robust group.

## Background

Frailty is a common and important geriatric syndrome associated with adverse outcomes, including hospitalization, disability, and mortality [1–4]. The prevalence of frailty in acute care settings varies from 33.5 to 68.5% [5]. Hospitalized frail older adults have higher rates of hospital-associated disability [6]. While the illness is treated during hospital admission, hospitalization itself may lead to limited functional recovery or even a new functional decline [7]. Moreover, hospitalized older adults who are discharged with new or additional disability in activities of daily living (ADL) have a poor long-term prognosis of functional recovery [8].

Acute Care for Elders (ACE) units are currently one of the strongest evidence that redesigned age-friendly care systems could improve functional outcomes and increase the likelihood of discharge to home [9–11]. ACE units provide age-friendly environments, patient-centered care, disability prevention and rehabilitation, medical care review, and early discharge planning [12]. The core components of ACE units are the interdisciplinary team-based care and comprehensive geriatric assessment, which are essential interventions in preventing and treating frailty [12, 13]. In a prospective controlled trial, older patients with severe frailty admitted to a comprehensive geriatric assessment (CGA) unit, one kind of ACE unit, were associated with a lower risk of functional decline, compared to those who received conventional acute care; however, the study measured ADL only before discharge and three months after that without considering the functional change during hospitalization [14].

The effect of functional disabilities recovery in relation to frailty status in geriatric care units remained undermined. One retrospective observational study in an acute geriatric ward showed that an increasing frailty status may be related to a lower functional recovery [15]; however, in a prospective case study in a geriatric evaluation and management (GEM) unit, which was a ward model of CGA with rehabilitation, frailer patients showed greater functional improvement [16]. Moreover, since admission to ACE units is usually short-term and their availability is limited, early identification of suitable hospitalized patients based on their frailty status is essential to maximize the effective use of the units. Thus, we aimed to determine whether frailty status is a determining factor for functional recovery (ADL) in an ACE unit.

## Methods

### Study design and setting

In this prospective observational study, patients from the acute geriatric ward of Hualien Tzu Chi Hospital between October 2019 to September 2020 were enrolled. The acute geriatric ward, an ACE unit, is the first and the only of its kind in eastern Taiwan and provides integrated care for older patients. The ACE unit provides age-friendly environments, patient-centered care, disability prevention and rehabilitation, medical care review, and early discharge planning. In addition to three geriatricians with expertise in the care of older adults, one trained geriatric resource nurse is involved in the team as a care manager. Extended team members include a clinical pharmacist, dietitian, social worker, as well as physical, occupational, and speech-language therapists. Multidisciplinary team meetings are conducted at least once weekly.

### Study population

We included patients aged ≥ 65 years who required acute inpatient medical care. Patients who were totally dependent for personal care before admission and those approaching the end of life were excluded. We also excluded those transferred to another unit within 24 h of admission, as they could not accept full ACE care and complete the assessments.

### Data collection

All the following data were collected by the trained geriatric resource nurse. We collected frailty status as well as the baseline clinical and demographic characteristics within 48 h of admission.

### Baseline clinical and demographic characteristics

We obtained information on age, sex, principal diagnosis, length of stay, body mass index (BMI), Lawton Instrumental Activities of Daily Living (IADL) [17], Mini-Mental State Examination (MMSE) [18], five-item Geriatric Depression Scale (GDS-5) [19], Charlson Comorbidity Index (CCI) [20], and potentially inappropriate medication (PIM) [21].

### Assessment of frailty

The FRAIL scale is a validated tool for frailty evaluation and is composed of the following five domains: fatigue, resistance, ambulation, illness, and loss of weight [22, 23]. Based on the total score, which ranges from 0 to 5 (1 point for each domain; 0 = best and 5 = worst), patients could be classified into three groups: robust (score = 0), prefrail (score = 1–2), and frail (score = 3–5). Moreover, this simple five-question scale is an optimal screening tool for clinicians to identify persons with frailty at risk of functional decline and mortality [23].

### Outcome measures

The associations of FRAIL scale categories (frail vs. robust; prefrail vs. robust) with functional change at admission and before discharge were examined based on ADL, short physical performance battery (SPPB), and grip strength.

#### ADL

Disability was assessed using the Barthel Index for ADL measurement [24, 25]. Basic ADL included the following ten items: feeding, bathing, grooming, dressing, bowel control, bladder control, toilet use, transfers, mobility on level surfaces, and stairs. The total ADL score was the sum of each item, which ranged from 0 to 100. A higher ADL score reflected a higher level of independence. We obtained ADL scores by interviewing the patients or their surrogates at three time points: baseline ADL (two weeks pre-admission), ADL at admission, and ADL before discharge.

#### SPPB

The SPPB is a simple measure of lower extremity performance using three-component tasks: static balance, gait speed, and chair stand [26]. Static balance was assessed with the patients standing in side-by-side, semi-tandem, and tandem positions; gait speed was evaluated with a 4-m walking test; chair stand was measured by the time needed to perform chair stand five times. Each component task was scored 0–4; the total score ranged from 0 (worst) to 12 (best).

#### Grip strength

Grip strength was assessed using a hand dynamometer (Smedley, TTM, Tokyo, Japan). The assessment was performed three times. All patients were instructed to hold the dynamometer with their dominant hand without squeezing their arms to their body in a standing or sitting position, depending on their ability [27, 28]. The best performance was used in the analysis.

### Statistical analysis

We divided the patients into three groups according to FRAIL score: robust (score = 0), prefrail (score = 1–2), and frail (score = 3–5). We used descriptive statistics to estimate the baseline clinical and demographic characteristics. One-way analysis of variance for continuous variables and chi-square test for categorical variables were used to compare population characteristics across three FRAIL groups. The difference in ADL, SPPB, and grip strength at admission and before discharge was determined using a paired sample t-test. We applied Pearson's correlation coefficient to evaluate the relationship between the length of stay and ADLs (including baseline ADL and ADL at admission). To assess whether frailty status was a predictor of functional recovery in an ACE unit, we employed multiple linear regression and examined the association between frailty status and the change in ADL (discharge ADL score minus admission ADL score) after adjusting for age, sex, CCI, BMI, MMSE, GDS-5, and PIM. We also applied multiple linear regression using frailty status, baseline ADL, length of stay, and principal diagnosis, as the independent variables for the ADL changes. A two-tailed probability value of < 0.05 was considered statistically significant. All statistical analyses were performed using the IBM SPSS Statistics for Windows, version 22 (IBM Corp., Armonk, NY, USA).

## Results

Of the 120 patients who were admitted to the ACE unit, five who were totally dependent for personal care and approaching the end of life and one who was transferred to another ward on the same admission date were excluded. The remaining 114 participants were classified into three groups by FRAIL score: robust (*n* = 28, 24.6%), prefrail (*n* = 57, 50%), and frail (*n* = 29, 25.4%). The mean age was 79.8 ± 8.1 years, and 77 (67.5%) were females. The three most common causes of admission were urinary tract infection (38.6%), pneumonia (10.5%), and gastric ulcer (8.8%). The average length of stay was 6.4 ± 5.6 days. Most patients had multimorbidity (mean CCI, 5.6 ± 1.8). A comparison of the baseline clinical and demographic characteristics of the patients in the robust, prefrail, and frail groups are shown in Table 1. Age increased with increasing frailty status of the patient (mean age: robust, 78.4 ± 8.4 years; prefrail, 78.8 ± 8.1 years; frail, 83 ± 6.9 years). Patients in the frail group were more dependent on baseline ADL (before admission) and IADL (at admission) than those in the prefrail and robust groups (mean baseline ADL: robust, 98.8 ± 3.5; prefrail, 84.3 ± 23.2; frail, 69.7 ± 29.5; mean IADL: robust, 6.5 ± 1.7; prefrail, 4.7 ± 3.1; frail, 2.2 ± 2.7). Regarding cognitive function and mood, the frail group had a significantly lower MMSE score and a higher GDS-5 score than the other groups. Length of stay was not associated with frailty status, baseline ADL and ADL at admission. There were 49 patients (43.0%) who reported a fall history one year before admission. 4 patients (3.5%) had delirium, and 2 patients (1.8%) had falls during hospitalization.

**Table 1:** Baseline clinical and demographic characteristics by frailty status

Variable	Robust	Prefrail	Frail	*P*-value
	*n* = 28	*n* = 57	*n* = 29	
Demographic data				
Age, years	78.4 (8.4)	78.8 (8.1)	83.0 (6.9)	0.04
Female, n (%)	18 (64.3)	39 (68.4)	20 (69.0)	0.91
BMI (kg/m^2^)	25.3 (3.7)	25.1 (4.9)	24.4 (3.3)	0.68
Clinical data				
ADL before admission	98.8 (3.5)	84.3 (23.2)	69.7 (29.5)	< 0.001
Lawton IADL	6.5 (1.7)	4.7 (3.1)	2.2 (2.7)	< 0.001
MMSE	21.3 (5.6)	18.9 (5.7)	16.6 (6.3)	0.01
GDS-5	0.4 (0.7)	0.8 (1.3)	1.6 (1.5)	0.002
PIM	0.4 (1.2)	0.4 (0.8)	0.5 (0.9)	0.91
CCI	5.4 (2.3)	5.4 (1.6)	6.1 (1.5)	0.17
Length of stay, days	7.3 (10.3)	5.9 (2.9)	6.6 (2.5)	0.55

*Note.* Data are presented as n (%) or mean (standard deviation). *BMI* Body mass index (calculated as weight in kilograms divided by height in meters squared), *ADL* Activities of daily living (measured by the Barthel Index; range, 0–100), *Lawnton IADL* Lawton instrumental activities of daily living (range, 0–8), *MMSE* Mini-mental state examination (range, 0–30), *GDS-5* Five-item geriatric depression scale (range, 0–5), *PIM* Potentially inappropriate medication, *CCI* Charlson comorbidity index (range, 0–37)

Table 2 shows the effect of the ACE unit on ADL, SPPB, and grip strength at admission and discharge. The mean admission ADL score was 60.3 ± 31.9, and the mean discharge ADL score was 83.7 ± 21.6. Paired sample t-test was used to compare the mean functional recovery between admission and discharge; the ADL was significantly different (mean ADL gain, 23.4; 95% confidence interval [CI], 19.7–27; *P* < 0.001). Regarding physical function, significant differences in SPPB (mean SPPB gain, 2.2; 95% CI, 1.7–2.7; *P* < 0.001) and grip strength (mean grip strength gain, 1.2; 95% CI, 0.5–1.8; *P* = 0.001) were found.

**Table 2:** Functional recovery between admission and discharge by frailty status

	Admission	Discharge	Difference	*P*-value
ADL				
Total (*n* = 114)	60.3 ± 31.9	83.7 ± 21.6	23.4 ± 19.7	< 0.001
Robust (*n* = 28)	83.4 ± 15.6	96.8 ± 6.4	13.4 ± 13.5	< 0.001
Prefrail (*n* = 57)	61.6 ± 31.3	85.6 ± 19.0	24.0 ± 20.7	< 0.001
Frail (*n* = 29)	35.5 ± 27.2	67.2 ± 25.8	31.7 ± 19.1	< 0.001
SPPB				
Total (*n* = 114)	5.6 ± 3.7	7.8 ± 3.4	2.2 ± 2.5	< 0.001
Robust (*n* = 28)	8.1 ± 2.9	9.8 ± 2.3	1.6 ± 2.4	0.002
Prefrail (*n* = 57)	5.5 ± 3.4	7.9 ± 3.5	2.4 ± 2.6	< 0.001
Frail (*n* = 29)	3.5 ± 3.4	5.8 ± 2.9	2.3 ± 2.4	< 0.001
Grip strength				
Total (*n* = 114)	17.7 ± 8.3	18.9 ± 7.6	1.2 ± 3.6	0.001
Robust (*n* = 28)	20.2 ± 5.8	21.1 ± 6.3	0.9 ± 3.1	0.13
Prefrail (*n* = 57)	17.5 ± 9.4	19.0 ± 8.5	1.5 ± 4.0	0.008
Frail (*n* = 29)	15.7 ± 7.4	16.5 ± 6.1	0.8 ± 3.0	0.39

*Note.* Data are presented as mean ± standard deviation. *ADL* Activities of daily living (measured by the Barthel Index; range, 0–100), *SPPB* Short physical performance battery (range, 0–12)

**Fig. 1:**
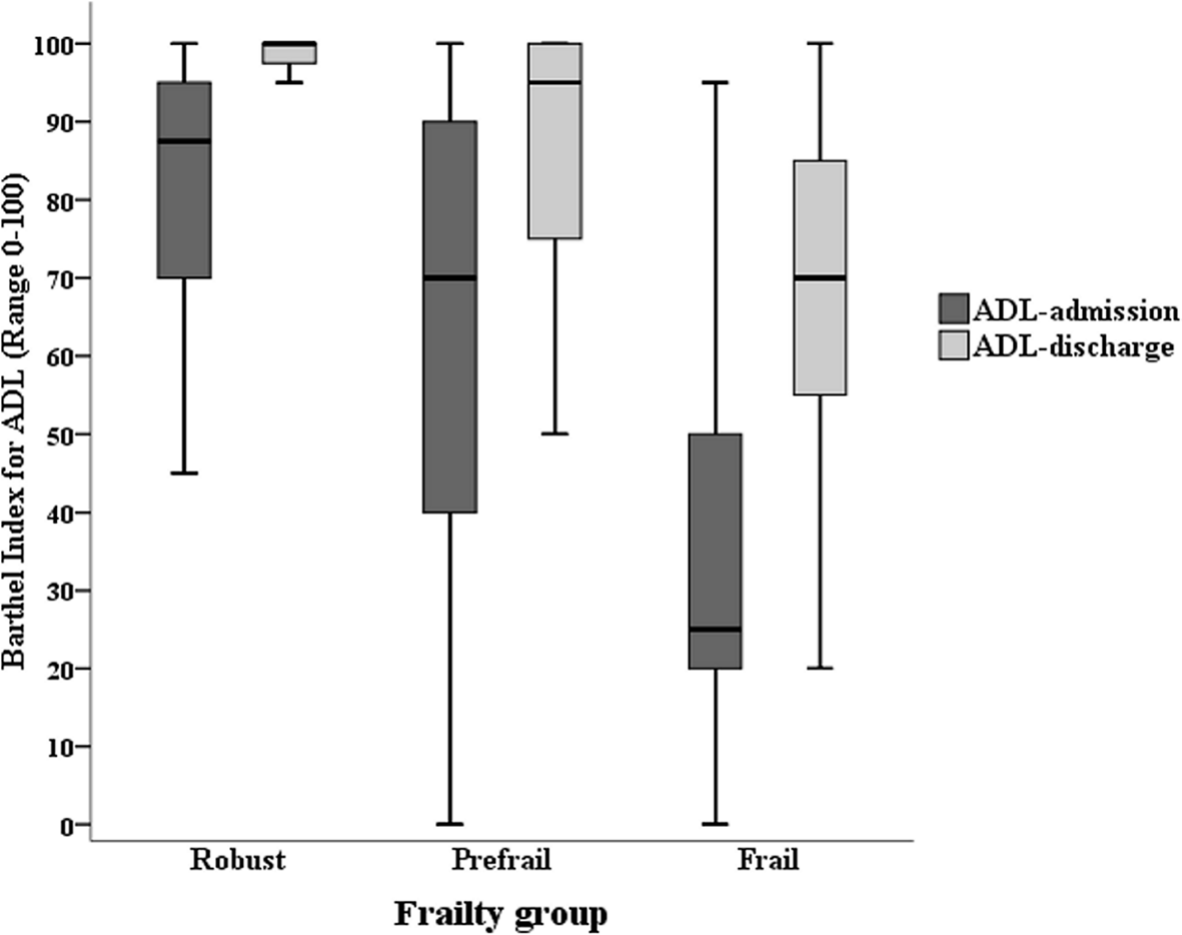
Admission and discharge Barthel Index for activities of daily living across frailty status. Abbreviations: ADL Activities of daily living

The effect of the ACE unit on ADL and SPPB was significant in the different frail groups. All groups showed improved ADL and SPPB at discharge. The frail group had the most significant change in mean ADL score (mean ADL gain, 31.7; 95% CI, 24.5–39; *P* < 0.001; Fig. 1), and only the prefrail group showed a significant change in mean grip strength (mean grip strength gain, 1.5; 95% CI, 0.4–2.5; *P* = 0.008).

Furthermore, multiple linear regression (Table 3) showed that prefrail and frail groups were significantly associated with ADL change between admission and discharge, after adjusting for age, sex, BMI, MMSE, GDS-5, PIM, and CCI, compared with the robust group (prefrail vs. robust: β = 9.0; 95% CI, 0.3–17.6; *P* = 0.04; frail vs. robust: β = 13.4; 95% CI, 2.7–24.0; *P* = 0.01). However, the frail group showed no association with ADL change compared with the prefrail group (frail vs. prefrail: β = 4.4; 95% CI, -4.4 to 13.2; *P* = 0.32). When frailty status, baseline ADL, length of stay, and principal diagnosis were used as independent variables, multiple linear regression still showed a significant association between frail group and changes in ADL (frail vs. robust: β = 12.6; 95% CI, 1.7–23.6; *P* = 0.03) (Table 4). Baseline ADL, hospital length of stay, and principal diagnosis were not significantly associated with ADL change between admission and discharge.

**Table 3:** Multiple linear regression using frailty status and CGA components as the independent variables for the ADL changes between admission and discharge

Variable	Unstandardized coefficients *B*	95% CI	*P*-value
Age, years	-0.02	-0.5 to 0.5	0.93
Sex (male vs female)	-0.2	-7.7 to 7.3	0.96
CCI	1.7	-0.4 to 3.9	0.12
**Frailty status**			
Prefrail vs robust	9.0	0.3 to 17.6	0.04
Frail vs robust	13.4	2.7 to 24.0	0.01
**CGA components**			
BMI (kg/m^2^)	-0.4	-1.3 to 0.5	0.37
MMSE	-0.6	-1.2 to 0.04	0.07
GDS-5	0.4	-2.4 to 3.3	0.75
PIM	2.5	-1.4 to 6.3	0.20

*Note. CGA* Comprehensive geriatric assessment, *ADL* Activities of daily living, *CI* confidence interval, *CCI* Charlson comorbidity index (range, 0–37), *BMI* Body mass index, *MMSE* Mini-mental state examination (range, 0–30), *GDS-5* Five-item geriatric depression scale (range, 0–5), *PIM* Potentially inappropriate medication

**Table 4:** Multiple linear regression using frailty status, baseline ADL, length of stay and principal diagnosis, as the independent variables for the ADL changes between admission and discharge

Variable	Unstandardized coefficients *B*	95% CI	*P*-value
Age, years	0.01	-0.5 to 0.5	0.96
Sex (male vs female)	-0.1	-7.9 to 7.8	0.98
CCI	1.9	-0.2 to 4.1	0.08
ADL before admission	-0.1	-0.3 to 0.04	0.13
Length of stay, days	-0.2	-0.8 to 0.5	0.58
**Frailty status**			
Prefrail vs robust	8.6	-0.5 to 17.8	0.06
Frail vs robust	12.6	1.7 to 23.6	0.03
**Principal diagnosis**			
Urinary tract infection vs other diagnoses^a^	-0.3	-8.4 to 7.9	0.95
Pneumonia vs other diagnoses^a^	-2.9	-15.4 to 9.6	0.65
Gastric ulcer vs other diagnoses^a^	4.5	-8.8 to 17.9	0.50

*Note. ADL* Activities of daily living, *CI* Confidence interval, *CCI* Charlson Comorbidity Index (range, 0–37)

^a^Patients with other diagnoses referred to all patients excluding those with the three most common principal diagnoses (urinary tract infection, pneumonia and gastric ulcer)

## Discussion

This prospective observational study found that older patients in the ACE unit may experience varying degrees of functional improvement across different frailty classifications based on the FRAIL scale. Compared to robust patients, those classified as prefrail and frail were associated with more functional recovery between admission and discharge.

Based on clinical evidence, ACE units could improve functional outcomes [12]; however, few studies have examined the effect of ACE units on functional outcomes in older patients with frailty [29]. Ekerstad et al. assessed frailty with the frail elderly support research group (FRESH) screening instrument, and the primary outcome was functional decline, which was evaluated by the ADL Staircase three months after discharge from the CGA unit. The CGA unit was essentially the same as the ACE unit. They found that most older patients with frailty in the CGA unit had no ADL change at the 3-month follow-up [14]. In the same clinical trial, older patients with frailty in the CGA unit improved significantly in all components of physical function, including handgrip strength, timed up-and-go test, and the 6-min walk test [30]. Our study found that older patients may benefit from the ACE unit care even during a short hospitalization period, and their ADL and physical function have improved.

This study was the first to use the FRAIL scale at admission to evaluate whether different baseline frailty status is a predictor of recovery of ADL function in an ACE unit. We confirmed that patients classified as prefrail and frail were associated with more functional improvement. The International Conference of Frailty and Sarcopenia Research has suggested the FRAIL scale as a screening tool for frailty [31]. It is a validated tool to predict disability with a similar extent to that of the Fried frailty phenotype and is more feasible than the Fried criteria in hospitalized older patients [32, 33]. In the acute hospitalization of geriatric patients with fracture and heart failure, previous studies showed that frailty, as assessed by the FRAIL scale, was associated with poor outcomes [34, 35].

Identifying the frailty status of patients in ACE units has below benefits that may contribute to the prevention or treatment of hospital-associated disability. During identifying the frailty status, the interdisciplinary team may be prompted with potential causes of frailty. For example, in patients with fatigue, in addition to treating acute illness, further investigation on possible related causes, such as postural hypotension, depression, hypothyroidism, vitamin B12 deficiency, and anemia, should be performed [36]. Moreover, patients classified as prefrail and frail, excluding those who are totally dependent for personal care [37, 38], have the potential for functional improvement, which is supported by the findings of our study and those of a previous study on GEM unit [16]. Furthermore, in an ACE unit, physical and occupational therapists provide multicomponent physical activity programs, dietitians recommend protein/caloric supplementation, and clinical pharmacists ensure that polypharmacy and medication-related harm are prevented. These strategies to avoid hospital-associated disability are also recommended as core interventions to prevent and treat frailty [31, 39]. Lastly, for areas with limited ACE resources, an ideal predictor of functional recovery, such as the FRAIL scale, may be needed when screening for older patients suitable for admission to ACE units.

Our study showed that lower extremity physical function, as measured by SPPB, improved in the three frail groups. Physical function was described as the capacity of an individual to perform physical ADL [40]. ADL evaluated by the Barthel Index [24], which is filled in by asking the patient or proxy, may be associated with recall bias. On the other hand, physical function tests, including handgrip strength, five-time chair standing, and 6-min walk test, could be performed objectively. These tests were found to be impaired in acutely hospitalized older patients with frailty [41]. Thus, our study used both ADL and physical function assessment to evaluate functional changes during hospitalization and for more reliable identification of the effect of the ACE unit.

The strengths of this study included its prospective design, which helped in the tracing of nearly all acutely hospitalized older patients in the ACE unit. Moreover, the measurements of frailty and functional outcomes using the FRAIL scale, Barthel Index, SPPB, and grip strength were feasible in the acute hospital setting, which in turn facilitated the identification of the association between frailty and functional change. In addition, we used different functional outcomes, including ADL and physical function, thereby making the evaluation of the effect of the ACE unit on older patients with different degrees of frailty possible.

This study has some limitations. First, our study excluded those patients with total dependence for personal care and approaching the end of life, which diminished the generalizability of our findings. However, patients with severe disabilities should be considered terminally ill and thus require care different from that in the frailty care spectrum; individual palliative care should be arranged [42]. Second, the baseline Barthel Index of the robust group was higher than that of the other groups; thus, a ceiling effect possibly made the robust group less sensitive to the effect of the ACE unit. Nevertheless, the inverse relationship between the magnitude of ADL change and the baseline ADL in our study could also be interpreted as a rate-dependency phenomenon; that is, the intervention response rate is highest among individuals with the lowest baseline values [43]. The result of the SPPB change was also in accordance with the ADL change. Lastly, the relatively small sample size and the absence of a control group might have limited the assessment of the effect of the ACE unit in different frail groups. Nonetheless, we identified the predictive effect of baseline frailty status in our study, which suggested that evaluating the frailty status at the beginning of hospitalization was essential in acute geriatric care.

## Conclusion

In conclusion, the ACE unit was associated with functional improvement in older patients with frailty. Among the different frail groups based on the FRAIL scale, the prefrail and frail groups were associated with more functional recovery during hospitalization. These findings open the possibility for a shift from the traditional disease-focused care to a redesigned age-friendly care system that recognizes frailty status as an important clinical predictor of functional improvement.

## Data Availability

The datasets generated and analysed during the current study are available from the corresponding author on reasonable request.

## Abbreviations

ACE: Acute Care for Elders
ADL: Activities of Daily Living
CGA: Comprehensive Geriatric Assessment
GEM: Geriatric Evaluation and Management
BMI: Body Mass Index
IADL: Instrumental Activities of Daily Living
MMSE: Mini-Mental State Examination
GDS-5: Five-item Geriatric Depression Scale
CCI: Charlson Comorbidity Index
PIM: Potentially Inappropriate Medication
SPPB: Short Physical Performance Battery
FRESH: Frail Elderly Support Research Group
